# Multi scale self supervised learning for deep knowledge transfer in diabetic retinopathy grading

**DOI:** 10.1038/s41598-025-85685-w

**Published:** 2025-09-30

**Authors:** Wadha Almattar, Saeed Anwar, Sadam Al-Azani, Fakhri Alam Khan

**Affiliations:** 1 Information and Computer Science Department, King Fahd University of Petroleum and Minerals, Dhahran, 31261 Saudi Arabia; 2 Department of Computer Science, Imam Abdulrahman Bin Faisal University, Dammam, 31441 Saudi Arabia; 3 SDAIA-KFUPM Joint Research Center for Artificial Intelligence, King Fahd University of Petroleum and Minerals, Dhahran, 31261 Saudi Arabia; 4 Interdisciplinary Research Center of Intelligent Secure Systems (IRC-ISS), King Fahd University of Petroleum and Minerals, Dhahran, 31261 Saudi Arabia

**Keywords:** Diabetic retinopathy grading, Self-supervised learning, Feature pyramid network, Vision transformer, CBAM, Classification and taxonomy, Experimental models of disease, Pathology

## Abstract

Diabetic retinopathy is a leading cause of vision loss, necessitating early, accurate detection. Automated deep learning models show promise but struggle with the complexity of retinal images and limited labeled data. Due to domain differences, traditional transfer learning from datasets like ImageNet often fails in medical imaging. Self-supervised learning (SSL) offers a solution by enabling models to learn directly from medical data, but its success depends on the backbone architecture. Convolutional Neural Networks (CNNs) focus on local features, which can be limiting. To address this, we propose the Multi-scale Self-Supervised Learning (MsSSL) model, combining Vision Transformers (ViTs) for global context and CNNs with a Feature Pyramid Network (FPN) for multi-scale feature extraction. These features are refined through a Deep Learner module, improving spatial resolution and capturing high-level and fine-grained information. The MsSSL model significantly enhances DR grading, outperforming traditional methods, and underscores the value of domain-specific pretraining and advanced model integration in medical imaging.

## Introduction

Diabetic retinopathy (DR) is a leading cause of vision loss worldwide, making early and accurate detection essential for effective treatment^1^. Automated deep learning systems for DR grading have shown significant potential in aiding clinicians with diagnosis. However, developing robust models for this task is challenging due to the complex, domain-specific nature of retinal images and limited availability of labeled medical data.

In medical imaging, transfer learning has been widely adopted, often leveraging models pre-trained on large-scale datasets like ImageNet^2^. These models, such as ResNet, Inception, DenseNet, EfficientNet, and Vision Transformers (ViTs), are fine-tuned to perform specific medical tasks. While this approach has proven effective in many computer vision tasks, its utility in medical imaging is not straightforward. The significant differences between medical and general images in datasets like ImageNet raise questions about the efficacy of such pre-trained models in the medical domain. Although pre-trained models typically outperform those trained from scratch in low-data scenarios, their weights do not always transfer effectively to medical tasks, limiting their potential in this specialized field^3^.

Recent advancements in self-supervised learning (SSL) have shown promise in addressing these limitations by enabling models to learn representations directly from the target domain^4–6^. Studies have demonstrated that SSL can outperform traditional supervised learning, particularly in medical imaging tasks where data is scarce and domain-specific. For example, self-supervised learning frameworks like SimCLR^7^, SwAV^8^, and DINO^9^ investigated in^10^, have been shown to produce richer and more transferable embeddings when applied to medical datasets, such as X-rays, dermatology images, and retinal fundus images. These approaches suggest that domain-specific pre-training and advanced model components are crucial for enhancing performance in medical imaging.

The backbone architecture in SSL plays a pivotal role in building scalable and effective solutions. Traditional CNN architectures, such as ResNet^11^, often struggle to scale with model and dataset size due to inherent inductive biases, including locality and weight sharing^12^. These biases lead CNNs to focus primarily on local regions within an image, potentially resulting in representations emphasizing local textures while neglecting global contextual information. This limitation is particularly significant in medical imaging, where global contextual understanding is often critical for accurate diagnosis.

In response to the challenges of diabetic retinopathy grading, we propose the Multi-scale Self-Supervised Learning (MsSSL) model, which integrates the strengths of Vision Transformers and Convolutional Neural Networks within a single self-supervised learning framework. Specifically, our approach incorporates the detailed multi-scale features extracted by a Feature Pyramid Network (FPN)^13^ with a ResNet backbone, along with self-attention mechanisms found in Vision Transformers^14^, which excel at capturing global, contextualized representations of input images. These fused features are then fed into the Deep Learner module, where they undergo systematic upsampling and refinement to enhance their spatial resolution, learn complex patterns and relationships within the data, and capture high-level and fine-grained information. The integration of these components results in more comprehensive and robust feature representations tailored specifically for accurate medical image diagnosis.

Our main contributions are as follows:

- Proposed a self-supervised learning framework named Multi-scale Self-Supervised Learning (MsSSL), that delivers robust performance, overcoming key limitations in supervised learning such as additional overhead, reliance on hybrid models, and the need for increased labeled samples, critical challenges in the medical field.
- Integrated two innovative modules, the Multi-scale Feature Extraction Encoder (MFEE) and the Deep Learner (DLe), into the self-supervised learning framework. These modules enhance feature representation by capturing both high-level and fine-grained features, addressing challenges such as false-negative issues in DR grading.
- Developed the ODM dataset, combining dual-view fundus images and DDR single-view samples to enrich SSL training, ensuring model generalization to various input types in downstream tasks.

In comparison to traditional transfer learning approaches that rely on weights transferred from large-scale datasets like ImageNet, the MsSSL model demonstrates significantly improved results in medical imaging tasks. This underscores the importance of domain-specific pretraining and the integration of advanced model components in enhancing the effectiveness of self-supervised learning approaches. The MsSSL model not only sets a new benchmark for representation quality and transferability in diabetic retinopathy grading but also offers a robust and adaptable solution tailored to the unique characteristics of medical imaging.

## Related work

### Transfer learning in medical imaging

Transfer learning is a key technique that leverages unlabeled data from the source or target domain, primarily using domain adaptation in semi-supervised learning^3^. It involves training a model on a large-scale dataset for one task and applying the pre-trained model to a new task^15^. Common models like ResNet50, Inception, and Vision Transformers (ViTs) are typically pre-trained on ImageNet^16^, the largest image dataset. However, medical images differ significantly from general images, raising questions about the effectiveness of ImageNet-pretrained models in medical imaging tasks^17–19^.

While pre-training on ImageNet enhances models performance in supervised learning, its utility in medical imaging, where data is scarce and difficult to annotate, is debated^2,3,20^. These pre-trained models often perform better than training from scratch but do not always transfer effectively to medical tasks. Furthermore, prior studies in supervised learning solutions tend to enhance performance by adding additional components to the models or employing ensemble techniques, which significantly increases model complexity^21^. Additionally, these solutions often require extending the number of training epochs, further escalating computational demands and training time.

Recently, self-supervised learning has gained traction for learning representations directly from the target domain^4–6,10,22,23^. Studies like^20^ demonstrated significant improvements with self-supervised learning (SimCLR) on X-ray and dermatology datasets. Similarly,^10^ showed that self-supervised pre-trained models like SimCLR, SwAV, and DINO produced richer embeddings, enhancing performance on medical datasets such as tumor detection, diabetic retinopathy, and chest X-rays.

ViTs, which capture global image features, offer an advantage in medical imaging over CNNs. When fine-tuned with domain-specific data, ViTs can outperform CNNs, improving self-supervised learning results^14,24^. This work explores combining ViTs and CNNs within a self-supervised framework to enhance feature learning for medical diagnosis and maintain no further model complexity in the downstream task.

### Multi-scale feature fusion

It is proved by^13^ that utilizing multi-scale features from different scales could effectively enhance the feature extraction during the learning process. Good feature extraction is essential in SSL learning; it is the critical point that alleviates representation learning when transferred to a specific downstream task. Studies confirm that high-level features contain more abstract semantic information, while low-level features contain more fine contextual information^25^. In supervised learning, either CNN-based or ViT-based, the feature representation is conveyed from a single level, which cannot accurately represent all image information. To overcome the issue with single-level features, multi-scale feature fusion has been shown to alleviate the representation, which is usually implemented by combinations, such as element-wise addition or channel-wise concatenation^26,27^. Other studies, instead, apply the fusion from different combination path^28,29^ or different models^10^.

**Figure 1:**
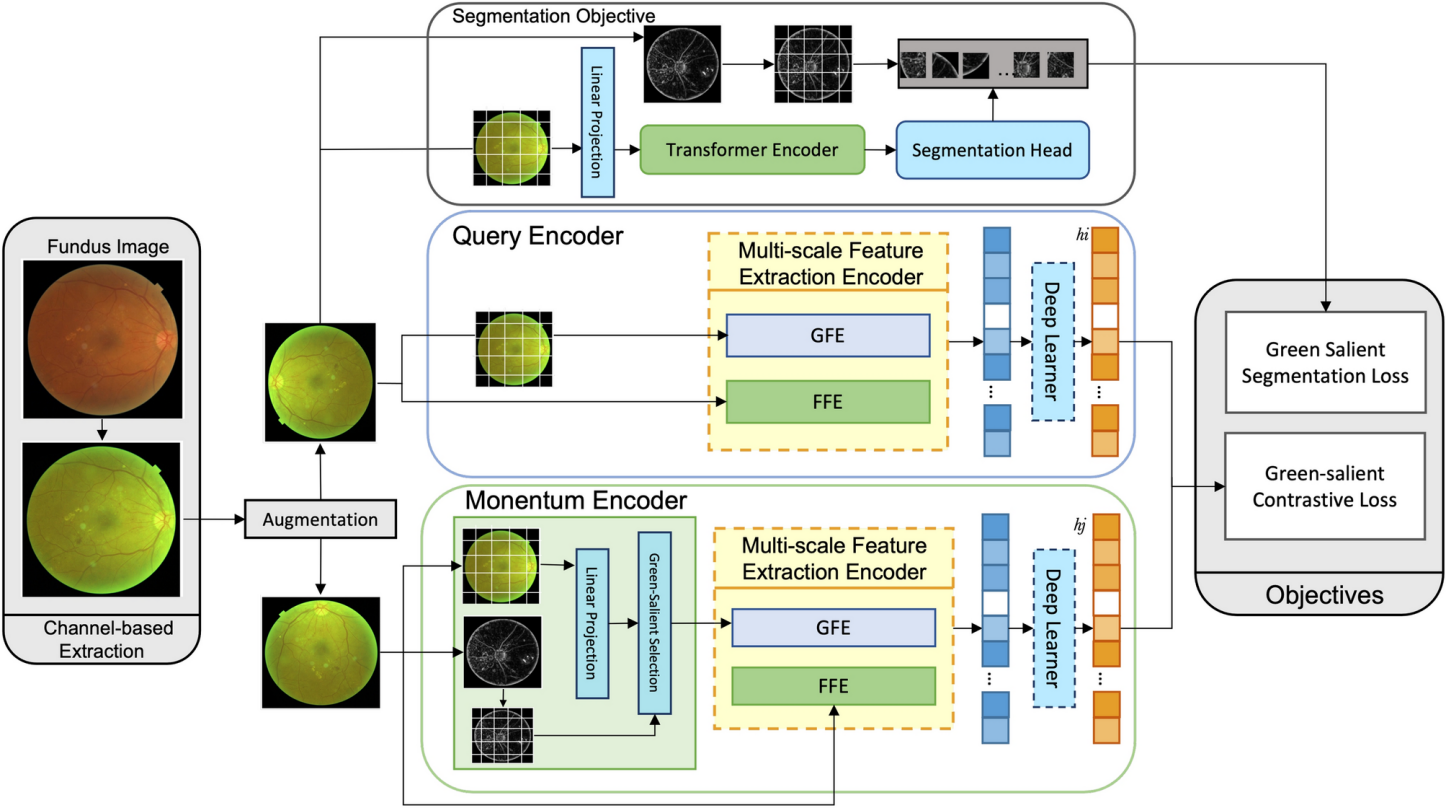
The proposed Multi-scale self-supervised learning (MsSSL) framework. GFE is the global feature extractor, and FFE is the fine-grained feature extractor. Tow objectives are calculated. *GFF* global feature extraction, *FFE* fine-grained feature extraction.

## Methodology

Contrastive self-supervised learning is a key area in self-supervised learning, focusing on learning representations by comparing image similarities. In this approach, an encoder produces embeddings that are either pulled closer together if similar or pushed apart if dissimilar, guided by a contrastive loss function. Typically, methods like SwAV and SimCLR treat each image as a unique class, training the model to distinguish it from others in the batch [16]. An alternative approach avoids instance discrimination by matching output features with those from a momentum encoder, thus generating target representations without negative pairs. Techniques such as BYOL, DINO, MoCo-v2, and MoCo-v3 fall into this category. Using momentum encoders to stabilize learning and improve the quality of representations. Our work aims to enhance this type of architecture for a more robust feature representation in diabetic retinopathy classification.

### MsSSL overall architecture

Our proposed architecture differentiates itself from SSL momentum encoder architecture by incorporating a multi-scale feature extraction encoder designed to extensively capture both high-level and fine- grained features a long side with the ViT encoder (Fig. 1). Additionally, after obtaining high spatial information from two distinct architecture bases, namely ViT and CNN-based extractors, we introduced a deep learner module to effectively learn these concatenated spatial features. We adopted a segmentation objective task, following the methodology outlined in previous work^22^, to capture subtle and local information. Building on our prior research^30^, we utilized the green channel instead of the RGB fundus image, with the entire training framework based on green-channel fundus images. Furthermore, we employed green salient detection from our earlier work to guide the momentum encoder towards salient regions in green-channel and to generate the ground truth for the segmentation objective.

### Multi-scale features extraction encoder

**Figure 2:**
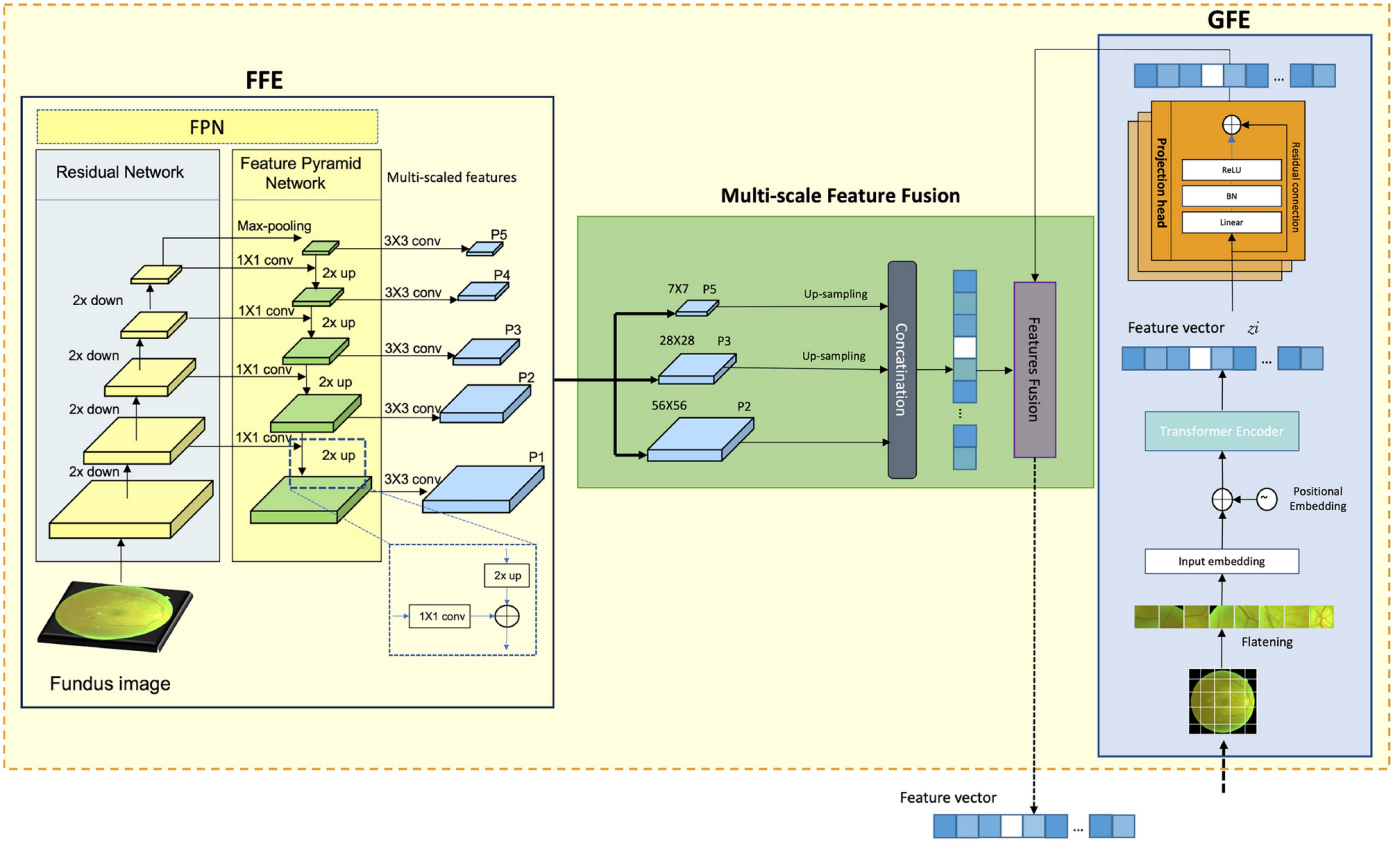
Multi-scale Feature Extraction Encoder architecture. *GFF* global feature extractor, *FFE* fine-grained feature extractor.

The multi-scale features extraction module is essential for capturing both high-level and fine-grained features, crucial for downstream tasks in SSL (Fig. 2a). This module combines the strengths of ViTs and CNNs, which extract both broad and detailed features through complementary methods. The module has three main components: the Global Feature Extractor (GFE), the Fine-Grained Feature Extractor (FFE), and the Multi-scale Feature Fusion.

**Global Feature Extractor (GFF)** This component uses a ViT to process fundus images by dividing them into patches, which are flattened and passed through a transformer encoder (Fig. 2a). The resulting feature vectors are then enhanced by a projection head and directed to the multi-scale feature fusion component.

**Fine-grained Feature Extractor (FFE)** The FFE utilizes a FPN based on ResNet to capture detailed image features. It constructs a pyramid of features at different scales, using P2, P3, and P5 levels (Fig. 2a,b). The P3 and P5 features are upsampled to match the P2 resolution before being sent to the fusion component.

**Multi-scale Feature Fusion** In this stage, the FFE’s extracted features are concatenated and fused with the GFE’s features using element-wise addition. This process integrates global and fine-grained features by element-wise addition, enhancing the overall representation (Fig. 2a). The final fused vector is then passed to the deep learner for further processing within the framework.

### Deep learner

**Figure 3:**
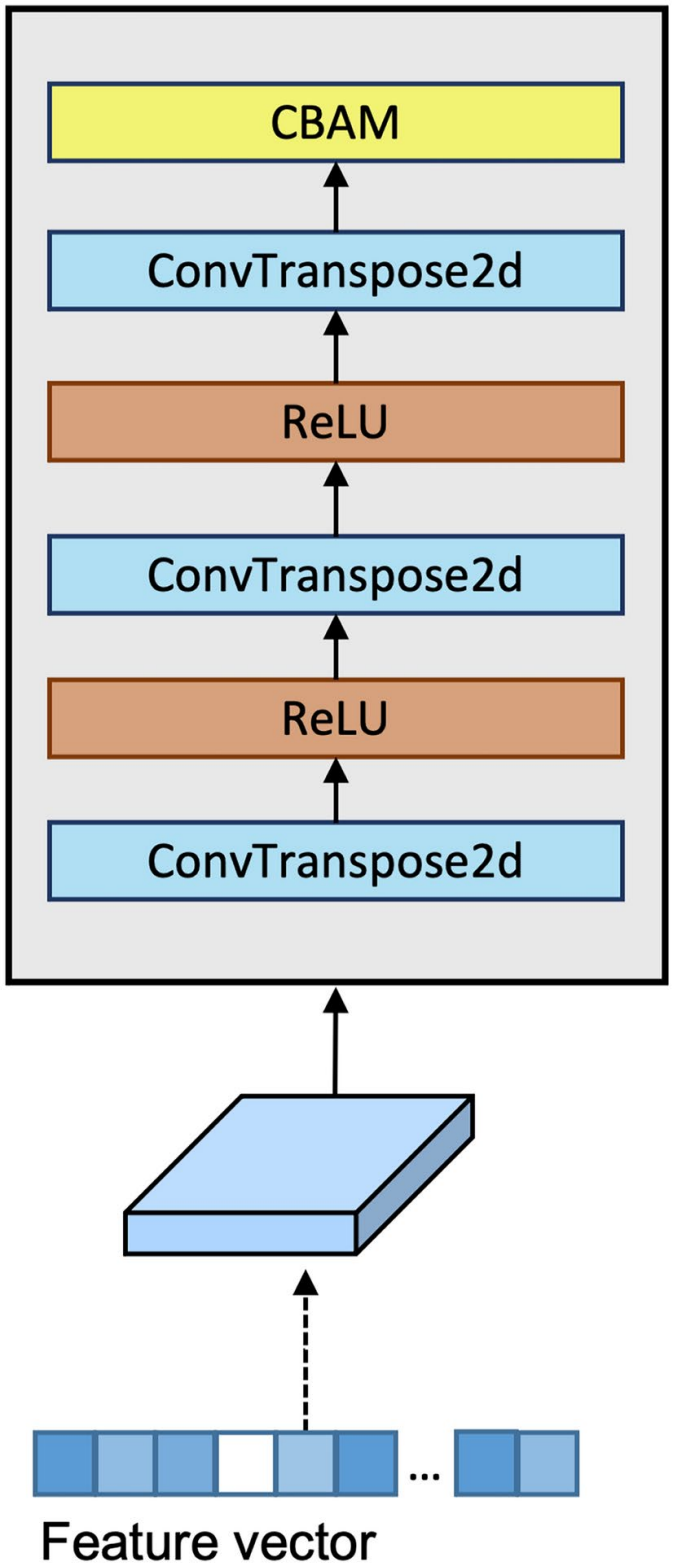
Deep Learner (DLe) architecture refines features through ConvTranspose2d layers with ReLU activations, ending with CBAM for enhanced feature attention and resolution.

The Deep Learner architecture refines fused features obtained from ViT and FPN. ViT captures high-level, global representations, while FPN, based on ResNet, extracts detailed, localized patterns (Fig. 2b). These features are combined through multi-scale feature fusion, then processed by the Deep Learner using ConvTranspose2d layers to incrementally upsample the data, reconstructing higher-resolution feature maps (Fig. 3). ReLU activations introduce non-linearity to capture complex patterns, and the final Convolutional Block Attention Module (CBAM)^31^ applies attention mechanisms to enhance significant features, improving the model’s representational power for complex image processing tasks.

## Experiment settings

### Supervised learning models

We evaluated a diverse set of CNN-based and Vision Transformer (ViT) architectures, all of which were pre-trained on the ImageNet dataset^32^. These models were adapted to our disease classification task using fine-tuning and transfer learning techniques.

**CNN-based models:** We employed several CNN-based models, each designed to address specific limitations of previous architectures through unique features. VGG-16, recognized for its straightforward design and reliable performance, uses a consistent structure of multiple convolutional layers with small 3x3 filters, followed by max-pooling layers^33^. ResNet models, particularly ResNet-18 and ResNet-50, introduced skip connections that help overcome the vanishing gradient problem, enabling the network to learn more complex patterns. The key difference between these models is their depth, with ResNet-18 featuring 18 layers and ResNet-50 featuring 50 layers^11^. EfficientNet-B5 improves performance by balancing the network’s depth, width, and resolution, offering better results but with increased computational demands^34^. Inception-V3, part of Google’s Inception series, employs a complex structure with inception modules that capture a broad range of features at different scales. This model also includes factorized 7x7 convolutions to reduce computational cost and an auxiliary classifier to enhance gradient flow and network regularization^35^.

**Transformer-based models:** We explored various transformer-based architectures, each tailored to harness the strengths of transformers in computer vision tasks. The base models, ViT-b-16 and ViT-b-32, utilize patch sizes of 16x16 and 32x32, respectively, and contain around 86 million parameters, showing competitive performance across numerous vision tasks. Larger models, such as ViT-L-16 and ViT-L-32, maintain the same patch sizes but incorporate additional layers and parameters, allowing them to capture more intricate patterns. The ViT-H-14 model, with a patch size of 14x14 and approximately 630 million parameters, represents one of the largest ViTs. All these models build upon the foundational ViT architecture, which includes components like image-to-patch transformation, linear projection, positional encoding, an encoder, and MLP layers^14^.

### Preprocessing

**Channel-based Extraction and Salient Detection.** Following our previous work^30^, we extracted the green channel from the fundus images, as it provides higher contrast for the detection of retinal lesions. We then applied salient region extraction to the green channel to emphasize the most critical areas of the images. In addition to these steps, we performed standard preprocessing procedures, including cropping to remove unnecessary borders and normalizing the pixel values to ensure consistent input for the model. These preprocessing steps were crucial in enhancing the quality of the input data, thereby improving the model’s ability to learn meaningful features from the images.

**Data Augmentation.** Under the self-supervised learning settings, we adopted the data augmentation strategy from SimCLR^7^ to enhance the variability and robustness of our training data. This approach includes several key operations: random cropping and resizing to fixed dimensions, ensuring positional invariance; horizontal flipping with a 50% probability to handle orientation changes; color jittering to adjust brightness, contrast, saturation, and hue, simulating various lighting conditions; Gaussian blurring with random kernel sizes to mimic out-of-focus scenarios; and normalization to align pixel value distributions with the pretraining dataset. These augmentations are essential for preventing overfitting and improving the model’s generalization by exposing it to diverse variations during training.

### Pretraining and evaluation datasets

For pre-training, we combined two distinct datasets: DDR^36^ without labels and the ODM-G (dual-view)^30^ dataset for SSL learning constructed in our previous work^30^. Integrating these datasets, collected from various locations and incorporating both single and dual views, enriched the data structures and formats, which is crucial for SSL. By exposing the model to samples of varying complexity, from simpler examples to more challenging ones, we aimed to improve its feature extraction capability. This approach allows the model to effectively generalize across different views and severity levels of diabetic retinopathy.

To evaluate the features learned during pretraining in our SSL setup, we conducted assessments on a benchmark dataset APTOS^37^. We randomly divide the dataset into three splits, 70%, 15%, and 15%, for training, validation, and testing, respectively.

### Evaluation protocols and matrices

To evaluate the representations learned by our SSL framework, we used fine-tuning and linear evaluation protocols. Fine-tuning assesses the transferability of representations by adapting the feature extractor for downstream tasks, while linear evaluation keeps the pretrained features fixed, focusing on the inherent quality and generalizability of the representations. For comprehensive evaluation, we report Kappa, Accuracy, and F1-score (weighted average), with an emphasis on the quadratic weighted Kappa metric due to its common use in DR classification studies. This metric, particularly effective in imbalanced multi-class tasks like DR grading, addresses class imbalance issues by evaluating performance across five distinct DR classes, as demonstrated in the Kaggle DR competition and APTOS study^37^.

## Results analysis and discussion

### Supervised transfer learning

A comparative analysis of supervised learning models on the APTOS dataset highlights the performance differences between models using random weights and those leveraging ImageNet-pretrained weights (Table 1). We trained ViT models (ViT-S-p16, ViT-S-p32) and compared them to CNN-based models commonly used in prior DR grading studies. This analysis investigates the effectiveness of supervised transfer learning using both pre-trained ImageNet weights and random initialization with models based on CNN and ViT architectures. The findings reveal the limitations of transfer learning from ImageNet to medical imaging tasks due to the domain gap between natural images and retinal fundus images.

**Figure 4:**
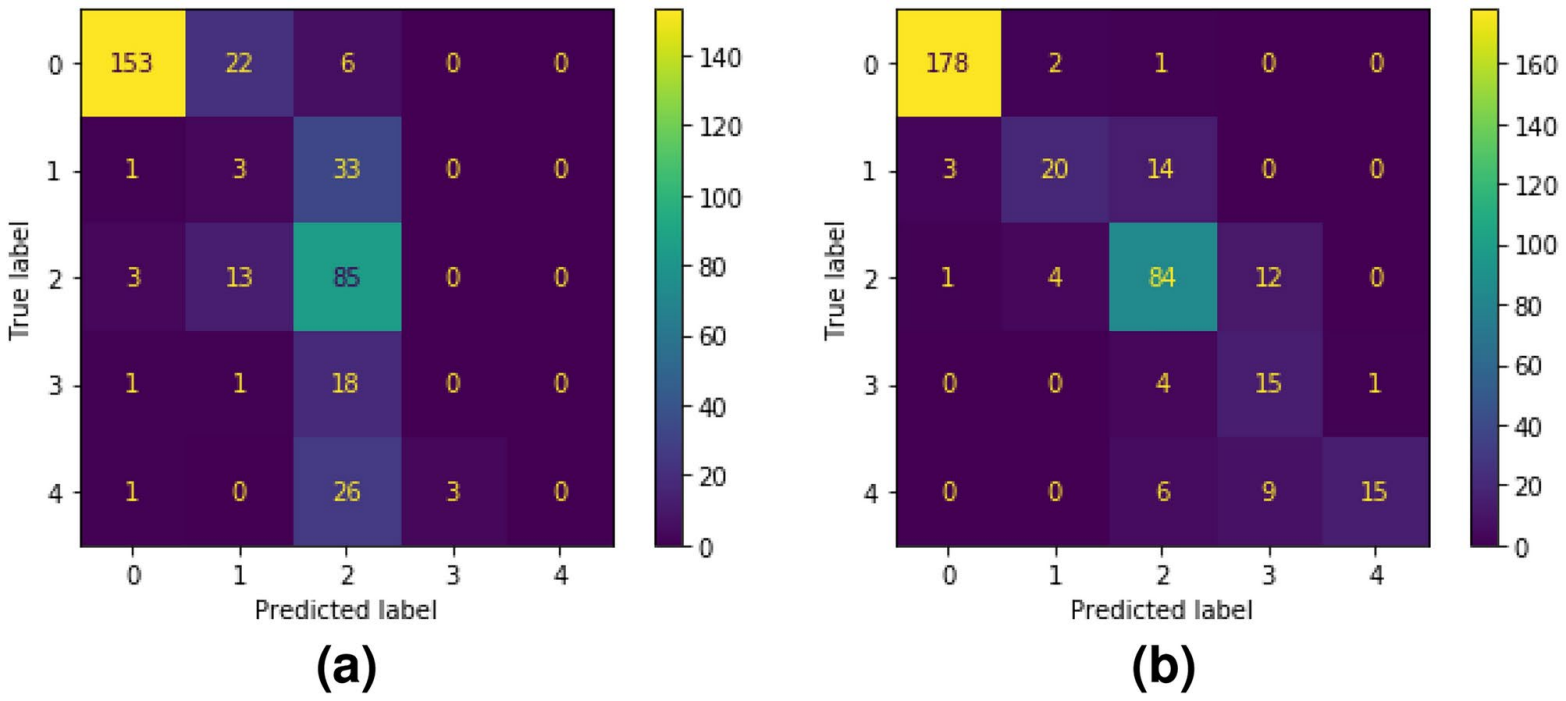
Confusion Matrices knowledge transferred from: (**a**) ImageNet, (**b**) MsSSL.

The results demonstrate that while transfer learning provides a useful starting point, it falls short of achieving accurate DR classification without extensive fine-tuning or domain-specific adaptations. ImageNet’s natural images lack the complex and nuanced features necessary for detecting DR, resulting in suboptimal recall scores, especially for minority classes (Fig. 4a). This highlights the need for domain-specific approaches. Our proposed SSL model, MsSSL, bridges this gap by enabling the effective transfer of domain-specific knowledge, achieving superior performance on the downstream DR task and addressing the inherent limitations of traditional supervised learning with ImageNet-pretrained weights.

**Table 1:** Comparison evaluation of supervised learning studies on APTOS dataset.

Supervised learning models	Weights	$\kappa$	Acc.%	F1%
ViT-S-p16	Random	0.7321	65.31	32.21
ViT-S-p32		0.6201	60.76	32.88
ResNet18^22^		0.5790	–	–
ResNet50^10^		0.5927	–	–
ViT-S-p16	ImageNet	0.8343	70.56	35.28
ViT-S-p32		0.7465	65.58	34.12
Hybrid Inception ResNet-v2^38^		–	82.18	–
E-DenseNet BC-169^39^		0.7001	80.22	–
E-DenseNet BC-201^39^		0.7301	82.20	–
ResNet50^10^		0.8057	–	–
Xception^40^		–	79.59	–
InceptionV3^40^		–	78.72	–
MobileNet^40^		–	79.01	–

### Self-supervised learning

**Representation transferability assessment.** Fine-tuning results on the APTOS dataset across various self-supervised learning (SSL) models presented in Table 2, emphasizing the importance of using in-domain weights, particularly in medical imaging.

Models pre-trained on ImageNet, such as S2C2L, show decent performance in general image analysis with kappa of 0.8907, accuracy of 83.08% and F1 of 82.27%. However, when applied to medical imaging, the benefits of using in-domain weights become clear. For instance, SimCLR pre-trained on EyePACS achieves a $\kappa$ value of 0.9034.

Our proposed MsSSL model, using ODM-DDR weights, outperforms others with a $\kappa$ value of 0.9324, accuracy of 84.55%, and an F1-weighted average of 84.57%, even when trained on a relatively small dataset compared to EyePACS. This demonstrates that domain-specific pre-training significantly enhances performance in medical imaging tasks by effectively capturing the nuanced features required for accurate diagnosis.

To provide a comprehensive evaluation of the model’s performance, we also calculated the F1-macro and F1-micro scores, which are 84.57% and 84.55%, respectively. The F1-macro score highlights the model’s ability to balance performance across all classes, ensuring fair representation even for minority classes, while the F1-micro score emphasizes the overall performance by considering each sample equally. Reporting both metrics ensures a thorough understanding of the model’s performance in handling imbalanced medical datasets, where class-level and overall performance are equally critical.

Moreover, MsSSL addresses the challenge of both false positive and false-negative which is a common issue in DR grading. The later which riskier because they can delay diagnosis and treatment, while false-positive is less riskier but leads to over-treatment or unnecessary follow-up. Notably, MsSSL shows a reduction in false negative compared to ViT-S16, where performance was limited by weights transferred from non-medical domains. This highlights the effectiveness of MsSSL’s self-supervised learning approach in improving classification accuracy for both majority and minority DR classes.

**Figure 5:**
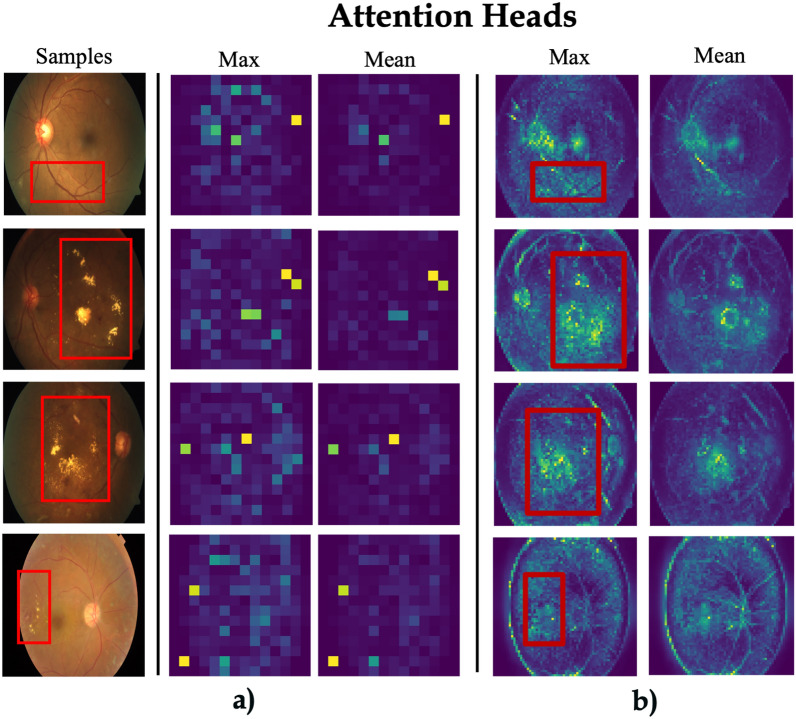
Self-attention map visualization of: (**a**) ViT-S16 where the knowledge transferred from ImageNet, (**b**) ViT-S16 where the knowledge transferred from MsSSL.

We present visualizations of self-attention maps of supervised and self-supervised ViT-S16. The fundus images are randomly sampled from the APTOS dataset, which was not used during pre-training. Recently, DINO^41^ demonstrated that the attention maps generated by self-distillation-based pre-trained ViTs capture semantic information from natural images. Following their approach to produce detailed attention maps, the images are resized to a higher resolution of 1024 $\times$ 1024, resulting in 64 $\times$ 64 patches with a patch size of 16 used as the input sequence. These images are then processed by self-supervised ViTs, and the self-attention maps from the final layer are displayed. We visualize the attention by averaging the normalized multi-headed self-attention maps for the global token, offering insights into how these models attend to different regions of the input. The visualization of ViT-S16 model, trained under a supervised learning regime, produces relatively sparse and less focused attention maps, with minimal emphasis on pathological regions. This scattered attention suggests that the model struggles to localize important features associated with the disease, which may reduce its effectiveness in medical diagnosis tasks. In contrast, the attention maps generated by ViT-S16 with MsSSL knowledge exhibit a clear advantage, as the model consistently highlights critical regions, including lesions and abnormal structures, across all the samples (Fig. 5).

**Table 2:** Comparative evaluation of fine-tuned SSL models on the APTOS dataset.

Model	Weights	$\kappa$	Acc.%	F1%
SwAV^10^	ImageNet	0.8293	–	–
SimCLR^10^		0.8264	–	–
S2C2L^42^		0.8907	83.08	82.27
DINO^9^		0.8365	–	–
DVME^10^		0.8242	–	–
SimCLR-RT^43^		–	83.1	–
SimCLR-FT^43^		–	83.32	–
MoCo-v2	EyePACS	0.9020	–	–
PCRL		0.8941	–	–
SimCLR		0.9034	–	–
MoCo-v3		0.8824	–	–
DINO^44^		0.8824	–	–
MAE		0.9034	–	–
MSN^44^		0.8887	–	–
**MsSSL(Ours)**	ODM-DDR	**0.9324**	**84.55**	**84.57**

Significant values are given in bold.

**Representation quality assessment.** Evaluating the quality of learned representations on the APTOS dataset through linear evaluation represented in Table 3, focusing on the importance of in-domain weights for medical image analysis. The proposed MsSSL model, using ODM-DDR weights, outperforms others with a $\kappa$ value of 0.8698, demonstrating the highest performance among the compared SSL models. Other models, such as SwAV, SimCLR, DINO, and DVME, which rely on ImageNet weights, report lower $\kappa$ values of 0.7617, 0.6989, 0.7790, and 0.8242, respectively. Models pre-trained on alternative datasets, like PCRL with EyePACS, achieve a $\kappa$ value of 0.7547. Additionally, DiRA and MoCo-v2 reach $\kappa$ values of 0.6980 and 0.8182, while MoCo-v3 and DINO score 0.8159 and 0.8034, respectively. The MAE model also performs well, with a $\kappa$ of 0.8324. These results underscore the critical importance of using in-domain images for pretraining in medical image analysis. The superior performance of the MsSSL model indicates that domain-specific SSL approaches significantly enhance the quality of learned representations, improving model accuracy and reliability in medical datasets.

**Table 3:** Comparison results of linear evaluation on APTOS dataset.

Model	Weights	$\kappa$
SwAV^10^	ImageNet	0.7617
SimCLR^10^		0.6989
DINO^10^		0.7790
DVME^10^		0.8242
DiRA	EyePACS	0.6980
MoCo-v2		0.8182
PCRL		0.7547
MoCo-v3		0.8159
DINO		0.8034
MAE		0.8324
**MsSSL(Ours)**	ODM-DDR	**0.8698**

Significant values are given in bold.

## Ablation studies

To validate our MsSSL framework, we conducted ablation studies on the APTOS dataset to assess each component’s impact on diabetic retinopathy (DR) grading. Trained for 300 epochs, we evaluated various backbone architectures, feature fusion methods, and attention mechanisms. The best-performing configuration used ResNet50 as the FPN backbone, ViT-S-p16 as the Vision Transformer, element-wise addition for feature fusion, and CBAM. This setup optimized key metrics, including Kappa score, accuracy, and F1 score, confirming the importance of careful module selection for optimal performance in medical image analysis (Table 4).

**Table 4:** MsSSL best settings.

Component	Setting
Saliency Detection	Fine grained
FPN Backbone	ResNet50
ViT Backbone	ViT-S-p16
Fusion	Addition
DLe Layers	Conv2DTransposed
DLe Attention	CBAM at the end

This ablation analysis not only highlights the importance of each module within the MsSSL framework but also reinforces the necessity of carefully selecting and combining these components to achieve optimal performance in medical image analysis. The extended training of 300 epochs allowed the model to fully explore the feature space, resulting in a robust transfer of knowledge that was effectively fine-tuned for the APTOS dataset, enhancing the model’s accuracy in detecting diabetic retinopathy.

### FPN-backbone

To evaluate the impact of different Feature Pyramid Network (FPN) backbones on the performance of the MsSSL model, we compared ResNet18 and ResNet50. FPN enhances feature extraction by integrating multi-scale features, essential for diabetic retinopathy (DR) grading, where both global and fine-grained details are important.

As shown in Table 5, ResNet50 outperformed ResNet18 across all metrics. ResNet50 achieved a Kappa coefficient of 0.9324, accuracy of 84.55%, and an F1 score of 84.57%, while ResNet18 reached a Kappa score of 0.9216, accuracy of 82.38%, and an F1 score of 69.49%. The deeper architecture of ResNet50 enables more robust feature extraction, capturing finer details crucial for detecting subtle variations in DR severity. These results highlight the importance of deeper backbones like ResNet50 in medical image analysis, where improved feature representation translates into more accurate predictions, especially for complex tasks like DR grading.

**Table 5:** MsSSL model with different FPN backbones.

FPN backbone	$\kappa$	Acc.%	F1%
ResNet18	0.92216	82.38	69.49
ResNet50	**0.93240**	**84.55**	**84.57**

Significant values are given in bold.

**Table 6:** MsSSL model with different ViT models backbones.

ViT backbone	$\kappa$	Acc.%	F1%
ViT-S32	0.9089	79.67	65.17
ViT-B16	0.9300	82.66	69.67
ViT-B32	0.9102	78.86	64.52
ViT-S16	**0.9324**	**84.55**	**84.57**

Significant values are given in bold.

### ViT variants as backbone

The choice of Vision Transformer (ViT) backbone significantly influences the performance of the MsSSL model, affecting its ability to learn and generalize from medical imaging data. We evaluated several ViT variants, including ViT-S16, ViT-S32, ViT-B16, and ViT-B32, to explore the impact of patch size (16x16 vs. 32x32) and model scale (Small vs. Base) on diabetic retinopathy (DR) grading, as shown in Table 6.

Among these, ViT-S16 outperformed the others, achieving a Kappa coefficient of 0.9324, accuracy of 84.55%, and an F1 score of 84.57%. The smaller 16x16 patch size combined with the simpler ViT-S architecture provided an optimal balance between feature detail and model complexity. This fine-grained feature extraction is crucial for medical imaging tasks, where subtle visual differences can affect diagnosis.

In contrast, variants like ViT-B32 and ViT-S32, using 32x32 patches, performed worse, with Kappa scores of 0.9102 and 0.9089, respectively, likely due to their reduced ability to capture detailed features. These results suggest that smaller patches and simpler architectures, as in ViT-S16, offer the best trade-off between accuracy and computational efficiency for DR grading.

### Feature fusing approaches

The choice of feature fusion methods is crucial for the MsSSL model’s performance, particularly in integrating multi-view features for accurate diabetic retinopathy (DR) grading. We experimented with three fusion methods: concatenation, element-wise multiplication, and element-wise addition (Table 7).

**Table 7:** MsSSL model with different fusion methods.

Fusion	$\kappa$	Acc.%	F1%
Concatenation	0.9198	81.84	68.19
Multiplication	0.9262	84.01	71.86
Addition	**0.9324**	**84.55**	**84.57**

Significant values are given in bold.

Element-wise addition delivered the best results, with a Kappa score of 0.9324, accuracy of 84.55%, and an F1 score of 84.57%. This suggests it effectively combines complementary information from the feature maps, enhancing the model’s ability to distinguish between DR stages. In contrast, element-wise multiplication, which restricts feature diversity, had the lowest performance with a Kappa score of 0.9034. Concatenation performed moderately with a Kappa score of 0.9198 but still lagged behind addition.

These findings underscore the importance of selecting the right fusion method, with element-wise addition proving to be the most effective for MsSSL, enabling better feature integration and superior model performance.

### Salient detection

Saliency detection is critical in medical image analysis, especially for diabetic retinopathy (DR) grading, where identifying relevant features can significantly improve model performance. We tested two methods within our MsSSL framework: spectral and fine-grained saliency detection.

**Table 8:** MsSSL model with different saliency detection methods.

Salient detection	$\kappa$	Acc.%	F1%
Spectral	0.9152	79.13	62.53
fine-grained	**0.9324**	**84.55**	**84.57**

Significant values are given in bold.

As shown in Table 8, fine-grained saliency outperformed the spectral method across all metrics, achieving a Kappa coefficient of 0.9324, accuracy of 84.55%, and an F1 score of 84.57%, compared to the spectral method’s 0.9152 Kappa and 79.13% accuracy. These results demonstrate that fine-grained saliency is more effective in capturing critical details, improving the model’s ability to classify DR stages accurately.

**Table 9:** MsSSL model with and without the Deep Learner (DLe) module.

Model	DLe	$\kappa$	Acc.%	F1%
MsSSL	$\times$	0.9260	82.92	69.21
MsSSL	$\checkmark$	**0.9324**	**84.55**	**84.57**

Significant values are given in bold.

### The impact of deep learner module

The integration of the Deep Learner (DLe) module into the MsSSL framework significantly enhances model performance. Without the DLe module, the model achieves a Kappa score of 0.9260, accuracy of 82.92%, and an F1 score of 69.21% (Table 9). However, incorporating the DLe module increases these metrics, improving the Kappa score to 0.9324, accuracy to 84.55%, and the F1 score to 84.57%. This improvement highlights the DLe module’s effectiveness in extracting more meaningful representations, particularly for challenging classes.

**Table 10:** Deep Learner (DLe) module with different layers design.

Layers	Kernel	$\kappa$	Acc.%	F1%
Conv1D	3$\times$3	0.9149	80.75	66.60
Conv2D	3$\times$3	0.9277	82.38	69.31
Conv2DTransposed	3$\times$3	0.9288	83.46	69.48
Conv2DTransposed + CBAM First	3$\times$3	0.9189	82.65	68.35
Conv2DTransposed + CBAM Last	3$\times$3	**0.9324**	**84.55**	**84.57**

Significant values are given in bold.

To optimize feature extraction, we explored five different configurations of the DLe module, focusing on various convolutional layers and the integration of Convolutional Block Attention Modules (CBAM), as shown in Table 10. The initial design using a 1D convolutional layer yielded a Kappa score of 0.9149 and accuracy of 80.75%, but struggled with complex spatial features. Switching to a 2D convolutional layer improved performance, with a Kappa score of 0.9277 and accuracy of 82.38%.

We further enhanced the model by incorporating Conv2DTransposed layers, increasing the Kappa score to 0.9288 and accuracy to 83.46%. Introducing CBAM after the Conv2DTransposed layers led to the best results, with a Kappa score of 0.9324 and accuracy of 84.55%. These findings underscore the importance of strategic layer design and attention mechanisms in optimizing feature extraction for diabetic retinopathy grading.

### The impact of the transferred weights vs MsSSL design

**Table 11:** The impact of the transferred weights vs MsSSL design.

Model	MFEE&DL	Dual-view	$\kappa$
MsSSL	$\times$	$\times$	0.8751
MSSSL	$\times$	$\checkmark$	0.8904
MSSSL	$\checkmark$	$\checkmark$	**0.9324**

Significant values are given in bold.

The findings in Table 11,show that both the transferred weights and the proposed MsSSL design significantly contribute to the model’s overall performance, with their combination yielding the best results.

## Conclusion

Training self-supervised models for medical diagnosis is challenging due to the complexity of medical data. Our proposed MsSSL model demonstrates superior performance on a small yet diverse dataset, due to its integration of dual- and single-view samples, a Multi-scale Features Extraction Encoder, and a Deep Learner, which together enhance feature representation.

Compared to models using ImageNet weights or random initialization, MsSSL achieves significantly better results, highlighting the value of domain-specific pretraining and advanced model components for medical imaging tasks. Its ability to effectively capture diverse features demonstrates its potential for generalization in medical image analysis.

While the MsSSL model sets a new benchmark for SSL pretraining, this study has limitations. The evaluation was conducted on a single dataset (APTOS), and future work should extend testing to diverse datasets and different demographic groups or imaging systems. Additionally, further investigation into optimal data augmentation techniques and multi-scale learning objectives could enhance the model’s performance in DR grading and other medical imaging tasks.

## Supplementary Information

Supplementary Information.

## Data Availability

The datasets supporting this study’s findings are publicly available: DDR^36^ and APTOS^37^. However, ODM dataset (dual-view fundus images) supporting this study’s findings are available from the corresponding author upon reasonable request.

## Code availability

Code will be publicly available on Github: https://github.com/Wadha-Almattar.
