# Supplementary material for: Multi scale self supervised learning for deep knowledge transfer in diabetic retinopathy grading
Source: Sci Rep. 2025 Sep 30;15:33742. doi: 10.1038/s41598-025-85685-w (PMC12485178; doi:10.1038/s41598-025-85685-w)

**Optic-disc Macula Dataset (ODM)**

# Dataset Description:

Our dataset, ODM, which stands for Optic-disc Macula dataset, is designed explicitly for self-supervised learning, and differs from the original dataset used for supervised learning (DRTiD and DeepDRiD). We created a novel dataset ODM by combining two datasets tailored for self-supervised learning

In contrast, combining datasets from different sources acquired from various sites can enrich diverse data structures and representations, which is the basis of learning in SSL. This fusion of diverse data can provide more comprehensive knowledge for the SSL model. Furthermore, it is crucial to provide SSL models with varying difficulty levels of samples, where easy examples can guide the model's initial learning, while harder ones can push its boundaries and enhance representation learning.

Our constructed ODM dataset comprises 5089 pairs of two-field fundus images, each representing a specific grade. The ODM dataset exhibits imbalances, similar to the original dataset and many benchmark datasets in supervised learning. The development of this extensive dataset and its variations addresses a significant gap in the study community and drives future progress in dual-view DR diagnosis research using the self-supervised learning paradigm.

# ODM Dataset:

Link to the dataset [ODM Dataset (Click here)](https://www.dropbox.com/scl/fo/kl96g0y7y2o24grza4r4a/AJHOsx9sGJfbaBfNQ3JdG-Q?rlkey=z79xbdvdoxbv97msjo2yhmlkc&st=fn16ujha&dl=0). The link provides only ODM-RGB used in this study.


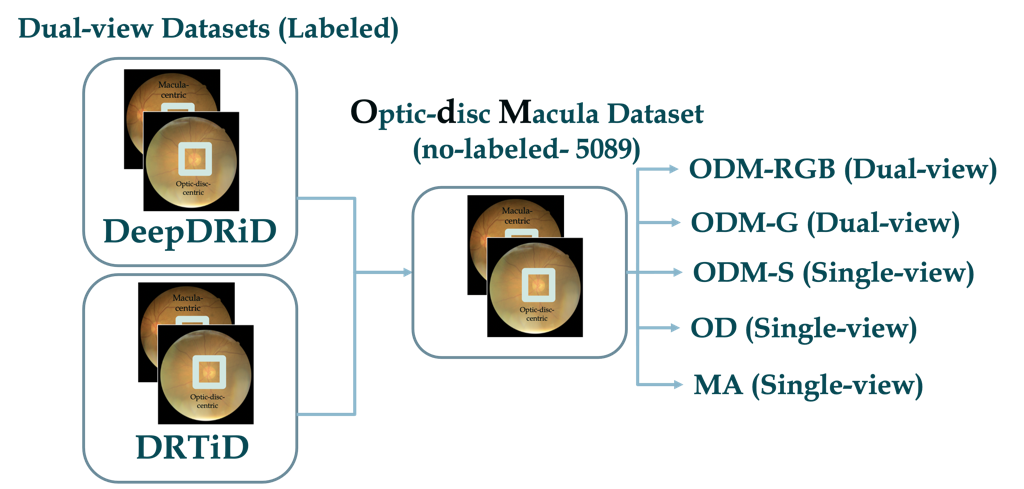

Supplement: Supplementary file 1 — Supplementary Information. [file 41598_2025_85685_MOESM1_ESM.docx]
